# Temporal Quantitative Proteomics Analysis of Neuroblastoma Cells Treated with Bovine Milk-Derived Extracellular Vesicles Highlights the Anti-Proliferative Properties of Milk-Derived Extracellular Vesicles

**DOI:** 10.3390/cells10040750

**Published:** 2021-03-29

**Authors:** Pamali Fonseka, Taeyoung Kang, Sing Chee, Sai V. Chitti, Rahul Sanwlani, Ching-Seng Ang, Suresh Mathivanan

**Affiliations:** 1 Department of Biochemistry and Genetics, La Trobe Institute for Molecular Science, La Trobe University, Melbourne, Victoria 3086, Australia; 19174837@students.latrobe.edu.au (T.K.); louischee@y7mail.com (S.C.); 19573709@students.latrobe.edu.au (S.V.C.); 19168073@students.latrobe.edu.au (R.S.); 2 Bio21 Institute, University of Melbourne, Melbourne, Victoria 3010, Australia; ching-seng.ang@unimelb.edu.au

**Keywords:** neuroblastoma, extracellular vesicles, bovine milk-derived extracellular vesicles chemotherapy, N-Myc

## Abstract

Neuroblastoma (NBL) is a pediatric cancer that accounts for 15% of childhood cancer mortality. Amplification of the oncogene N-Myc occurs in 20% of NBL patients and is considered high risk as it correlates with aggressiveness, treatment resistance and poor prognosis. Even though the treatment strategies have improved in the recent years, the survival rate of high-risk NBL patients remain poor. Hence, it is crucial to explore new therapeutic avenues to sensitise NBL. Recently, bovine milk-derived extracellular vesicles (MEVs) have been proposed to contain anti-cancer properties. However, the impact of MEVs on NBL cells is not understood. In this study, we characterised MEVs using Western blotting, NTA and TEM. Importantly, treatment of NBL cells with MEVs decreased the proliferation and increased the sensitivity of NBL cells to doxorubicin. Temporal label-free quantitative proteomics of NBL cells highlighted the depletion of proteins involved in cell metabolism, cell growth and Wnt signalling upon treatment with MEVs. Furthermore, proteins implicated in cellular senescence and apoptosis were enriched in NBL cells treated with MEVs. For the first time, this study highlights the temporal proteomic profile that occurs in cancer cells upon MEVs treatment.

## 1. Introduction

Extracellular vesicles (EVs) are membranous nanoparticles that are released from various cell types under physiological and pathological conditions [1,2,3]. EVs are heterogeneous in size and their lipid, protein and nucleic acid content often reflect the cells of origin [3,4,5]. Hence, EVs, through transferring the molecular cargo, play an important role in cell–cell communication and thereby can potentially modulate various signalling pathways in the recipient cells [3,6,7,8]. EVs are detected in various biological fluids including plasma, saliva and milk [9,10,11,12]. Milk is one of most highly consumed beverage due to its nutritional composition that aids in growth and development [13,14]. It has been established that milk contains high concentrations of EVs that are enriched with proteins and RNA that has the potential to elicit various signalling pathways in the recipient cells [15]. Recent studies have shown that milk-derived EVs (MEVs) can protect its cargo from harsh degrading conditions of the intestine and have proposed that dietary MEVs can be implicated in inter-individual, cross-species and kingdom communication [15,16,17,18,19,20]. In addition to their role in intercellular communication, bovine MEVs have also exhibited anticancer properties and can increase the efficacy of the anticancer drugs [21,22,23].

Neuroblastoma (NBL) is an extracranial solid tumour that arises from neuroblasts [24]. NBL patients are usually under the age of 5 [25]. When assigning treatments for NBL, age, biological feature and stage of the disease are considered [26,27]. One of the main risk factors is the amplification of the transcription factor N-Myc [28,29]. It is well established that N-Myc amplification is directly correlated with poor outcome and progression of the disease [30]. Even though the overall five-year survival rate of the NBL patients has improved recently, the survival rates of the children with high-risk NBL have not improved substantially [31]. Hence, there is an unmet need for better therapeutic avenues to treat high-risk NBL [32,33]. With the recent interest in the anti-cancer properties of MEVs, it is currently unknown whether MEVs have the potential to be utilised for NBL treatment.

In this study, bovine MEVs were characterized using nanoparticle tracking analysis and transmission electron microscopy. Incubation of MEVs with the N-Myc amplified NBL cells significantly attenuated the proliferation. Interestingly, combinatorial treatment of MEVs with doxorubicin sensitised the high-risk NBL cells. Comparative proteomic analysis of NBL cells treated with or without MEVs at various time points highlighted the depletion of proteins implicated in proliferation, cell cycle and wnt signalling pathway upon MEV treatment. Contrastingly, NBL cells treated with MEVs were enriched with proteins implicated in apoptosis and cellular senescence. Taken together, for the first time, this study summaries the temporal changes in the cellular proteome of cancer cells upon MEV treatment. The results obtained from this study suggest that combination treatment of bovine MEVs with chemotherapeutic drugs could be employed as a new treatment strategy to treat high-risk NBL.

## 2. Materials and Methods

### 2.1. Isolation of Milk-Derived EVs (MEVs) from Commercial Milk Samples

Five brands of commercial whole milk were purchased from local grocery stores in Melbourne, Australia. The milk samples were pooled in equal volumes and were subjected to centrifugation at 5000× *g* for 30 min at 4 °C. The floating milk fat layer was removed before centrifuging the samples consecutively at 12,000× *g*, 35,000× *g* and 70,000× *g* at 4 °C for 1 h each in order to remove the casein and other cell debris. The samples were subjected to final spin of 100,000× *g* using SW28 rotor (Beckman Coulter, Lane Cove West, NSW, Australia) at 4 °C for 1 h and the pellet (MEVs) obtained was washed with PBS. The MEVs were then filtered using a 0.2 µM syringe filters.

### 2.2. SDS-PAGE and Western Blotting

An equal volume/amount of protein samples was prepared in 4X sodium dodecyl sulfate (SDS) loading buffer containing 100 mM dithiothreitol (DTT) (Astral, NSW, Australia). Samples were then subjected to denaturation by heating at 95 °C for 2 min. The denatured samples were run on a NuPAGE^®^ 4–12% Bis-Tris precast gel (Life Technologies, Mulgrave, VIC, Australia) at 150 V for 1 h in the presence of NuPAGE^®^ MES SDS Running Buffer (Life Technologies, Mulgrave, VIC, Australia). Proteins were then transferred to mini nitrocellulose membranes using iBlot dry blotting system (Invitrogen™, Mulgrave, VIC, Australia) for 7 min at 20 V. The membrane was then blocked with skim milk 5% (*w/v*) in Tris-buffered saline with 0.05% (*v*/*v*) Tween 20 (TTBS) for 45 min. The membrane was washed with TTBS three times (10 min each) and probed with required primary antibodies at 1:1000 dilution overnight at 4 °C. The blot was then washed three times over 30 min with TTBS and then subsequently with appropriate IRDye (LI-COR^®^, Lincoln, NE, USA) conjugated secondary antibodies (1:10,000) for 1 h at room temperature. The probed blot was then washed three times with TTBS (10 min each). The protein bands were visualized using ODYSSEY Clx (LI-COR^®^, Lincoln, NE, USA).

### 2.3. Nanoparticle Tracking Analysis (NTA)

NanoSight N300 (Malvern Instruments, Malvern, UK) was used to visualize and analyse the size distribution of a population of MEVs. Sample chamber was monitored with the use of a 405 nm laser. MEV aggregates were separated using needle and syringe while simultaneously injecting to the NanoSight sample cubicle. The video frame rate used was 30 frames per second with the camera level at 7, detection threshold at 10 and flowrate at 50. Temperature was maintained at 25 °C. NanoSight NTA3.2 software was used for data analysis.

### 2.4. Transmission Electron Microscopy (TEM)

JEM-2010 transmission electron microscope (, 80 kV, JEOL, Tokyo, Japan) was employed to examine 0.2 mg/mL MEVs. The samples were fixed in mesh carbon-layered copper grids (400) for 2 min and the surplus preparations were drained using blotting paper. Samples were then stained with 10 µL of 2% (*w/v*) uranyl acetate solution (Electron Microscopy Services) before imaging.

### 2.5. Cell Culture

The NBL cell line, SK-N-BE2 (ATCC, Virginia, USA) and the colon cancer cell line C26 (gifted by Prof. Hoogenraad) were cultured in 150 cm^2^ tissue culture flask (BD Falcon™) in Dulbecco’s Modified Eagle Medium (DMEM) and Roswell Park Memorial Institute (RPMI) (GIBCO, Life Technologies) medium, respectively. The medium was supplemented with 10% (*v*/*v*) fetal calf serum (FCS) (GIBCO, Life Technologies) and 100 units/mL of penicillin-streptomycin (GIBCO, Life Technologies). The cells were incubated at 37 °C with 5% CO_2_ and were treated with 100 µg/mL of bovine MEVs. The cells were lysed using SDS lysis buffer (2% (*w*/*v*) SDS, 125 mM Tris-HCl pH 7.4, 12.5% (*v*/*v*) glycerol, and 0.02% (*w*/*v*) bromophenol blue) at 24, 48 and 72 h.

### 2.6. Isolation of EVs from C26 Colon Cancer Cell Line

When C26 cells reached 70–80% confluence, the cells were washed with phosphate-buffered saline (PBS) and supplemented with RPMI media containing EV depleted FCS. After 24 h of incubation, the conditioned media was collected and subjected to differential centrifugation (500× *g* for 10 min, 2000× *g* for 20 min and 10,000× *g* for 30 min). The supernatant was then subjected to ultracentrifugation at 100,000× *g* for 1 h at 4°C. The pelleted EVs were washed in PBS and stored in –80 °C until further use.

### 2.7. In Gel Digestion

Equal protein amount (30 µg) of cell lysates were separated using SDS-PAGE at 150 V. For visualization, separated protein bands were stained with Coomassie Brilliant Blue stain. The gel bands were extracted using scalpel blades. The bands were then reduced, alkylated and trypsinized as previously described [34,35]. Briefly, 10 mM DTT (Bio-Rad, Hercules, CA, USA) was used for reduction, 25 mM iodoacetamide (Sigma) was used for alkylation and the gels were trypsinized using 150 ng of trypsin (Promega). The tryptic peptides were extracted using 50% (*w*/*v*) acetonitrile and 0.1% (*v*/*v*) trifluroacetic acid.

### 2.8. LC-MS/MS

LTQ Orbitrap Velos (Thermo Scientific, Waltham, MA, USA) was employed when conducting LC-MS/MS. The nanoLC system was equipped with an Acclaim Pepmap nano-trap column Dionex–C18, 100 Å, 75 μm × 2 cm) and an Acclaim Pepmap RSLC analytical column (Dionex-C18, 100 Å, 75 μm × 15 cm) coupled with a nanoelectrospray interface. The volume loaded onto the enrichment (trap) column of peptide mix was 1 µL for each sample run. Before switching in-line with the analytical column, the isocratic flow of 3 µL/min of 3% (*v*/*v*) acetonitrile containing 0.1% (*v*/*v*) formic acid for 4 min was used. Volume of 0.1% (*v*/*v*) formic acid (solvent A) and 100% (*v*/*v*) acetonitrile 0.1% (*v*/*v*) formic acid (solvent B) were eluents used for the liquid chromatography. Prior to the final 15 min of 85% B, a gradient of 3% B to 8% B for 1 min, 8% B to 35% B for 30 min, 35% B to 85% B in 5 min was employed. The LTQ Orbitrap Velos mass spectrometer was operated with nano ESI spray voltage of +1.6 kv, capillary temperature of 250 °C and S-lens RF value of 60% in the data-dependent mode. The *m*/*z* change range of 300–2000 Da was accepted in the FT mode. A resolution of 30,000 after amassing to 1 × 10^6^ where all spectra were acquired in positive mode. Expected maximum accumulation was 500 ms and the 20 abundant precursor ions above 2 charged states were segregated at a target value of 1000. The set standard parameters were normalized collision energy of 30, activation Q of 0.25 and activation time of 10 ms. The applied dynamic exclusion settings were of 2 repeat counts over 30 s and exclusion duration of 70 s.

### 2.9. Identification of Proteins

The parameters used in generating the peak lists were a minimum mass of 300, a maximum mass of 5000, intermediate scans of 200, a minimum group count of 1, a total ion current of 100 and 10 peaks minimum. Extract-MSn as part of Bioworks 3.3.1 (thermo Scientific) was used. To enable MASCOT searches, each peak list obtained from LC-MS/MS runs was merged into a single mascot generic format. The Human RefSeq protein database [36] was employed when searching the LC-MS/MS spectra. Search parameters include fixed modification (carboamidomethylation of cysteine; +57 Da), variable modifications (oxidation of methionine; +16 Da), three missed tryptic cleavages, 20 ppm peptide mass tolerance and 0.6 Da fragment ion mass tolerance. If the ion score was greater than the identity score, the peptide identifications were deemed significant. These significant protein identifications, with a false-discovery rate less than 1%, contained at least 2 unique peptide identifications.

### 2.10. Label-Free Spectral Counting

The relative protein abundance between samples was obtained by estimating the ratio of normalized spectral counts (Rsc) as previously described [37]. When RSc is less than 1, the negative inverse RSc value was used

Where

Rsc = [(sY + c)(TX – sX + c)/(sX + c)(TY – sY + c)].

s = Significant MS/MS spectra for protein A.

T = Total number of significant MS/MS spectra in the sample.

c = Correction factor set to 1.25.

X and Y = Cell lysates.

### 2.11. MTS Assay

When seeding cells (5 × 10^3^ per 100 μL), 96-well plates were used. The cells were incubated for 24 h at 37 °C in 5% CO_2_ before further incubating with MEVs (100 µg/mL) or sonicated MEVs (100 µg/mL) or doxorubicin (1 µM) or a combination treatment of doxorubicin and MEVs or sonicated MEVs. The plates were then read for 0 h and every 24 h in the presence of 20 µL of 3-(4,5-dimethylthiazol-2-yl)-5-(3-carboxymethoxyphenyl)-2-(4-sulfophenyl)-2H-tetrazolium (MTS) solution (Phenazine methosulfate (PMS) reagent (Sigma Life Science^®^, NSW, Australia) in DPBS and CellTiter 96^®^ AQuueous MTS reagent powder (Promega, Alexandria, NSW, Australia) according to the manufacturer’s instructions). The plates were then incubated for 1.5 h after the addition of MTS solution. Plates were read using the SpectraMaxM5 multi-mode microplate reader (Molecular Devices) and the absorbance wavelengths used were 490 nm and 630 nm.

### 2.12. FACS Cell Death Assay

An equal number of SK-N-BE2 cells were seeded in 24-well plates before treating the cells with 100 µg/mL of MEVs or sonicated MEVs or EVs isolated from C26 cells. The cells were incubated with or without doxorubicin (1 µM) for 48 h at 37 °C and followed by staining of propidium iodide (PI) buffer before subjecting to Fluorescence-activated cell sorting (FACS) (FACS CANTO II (BD Biosciences, North Ryde, NSW, Australia) cell death analysis. Sonicated MEVs were prepared by sonicating the EV samples at 60% amplitude for 6 cycles of 30 s on/off for 3 min. At each 3 min interval, the samples were allowed to cool in ice for 2 min. The Sonicator Vibra-Cell (Sonics & Materials, Newtown, CT, USA) was employed for this process.

### 2.13. Bioinformatics and Statistical Analysis

The FunRich [38,39] analysis tool was used to generate the Venn diagrams, heatmaps and enrichment analysis. The statistical significance of an experiment was analysed by student *t*-test and one-way analysis of variance (ANOVA) with the Turkey–Kramer multiple comparison post-hoc test using GraphPad Prism 8 software.

## 3. Results

### 3.1. Isolation and Characterization of Bovine Milk-Derived Extracellular Vesicles

**Figure 1:**
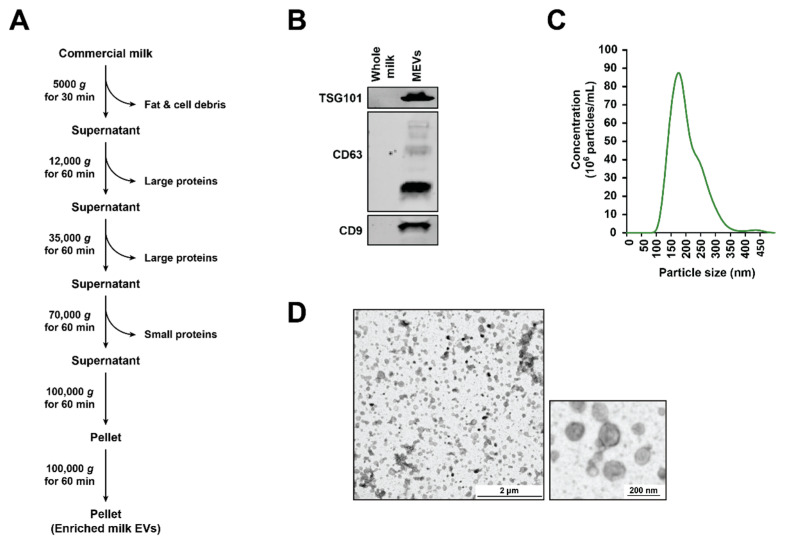
Characterization of milk-derived extracellular vesicles (MEVs). (**A**) Schematic diagram of MEVs isolation. (**B**) Western blotting analysis of 30 µg of MEVs and whole milk probed with TSG101. (**C**) Nanoparticle tracking analysis of MEVs with the highest peak at 170 nm. (**D**) Transmission electron miscopy (TEM) images suggested the presence of vesicles in the preparation.

EVs were isolated from a pooled mixture of commercially available whole-milk samples by differential centrifugation followed by ultracentrifugation as described previously [40]. The resulting EV pellet was washed with PBS, filtered with 0.2 µM filter and was referred to as MEVs (Figure 1A). Next, to confirm the presence of EVs, the samples were subjected to Western blotting analysis (Figure 1B). Compared to the whole milk samples (30 µg), equal protein amounts of the MEVs were enriched with TSG101, CD63 and CD9. Consistent with the previous studies, Alix was of low abundance in the isolated MEVs samples [40]. In order to characterize the isolated MEVs biophysically, a Nanoparticle tracking analysis (NTA) was carried out (Figure 1C). The highest peak was detected at 170 nm and vesicles up to 350 nm were identified suggesting that the isolated MEVs were heterogenous. Furthermore, transmission electron microscopy (TEM) was carried out to examine the morphology of the MEVs (Figure 1D). The MEVs exhibited a heterogeneous population of vesicles that were approximately of 50–250 nm in diameter. Taken together, these results confirm the isolation of MEVs from bovine milk.

### 3.2. Bovine Milk-Derived Extracellular Vesicles Attenuates the Metabolic Activity of Aggressive Neuroblastoma Cells

**Figure 2:**
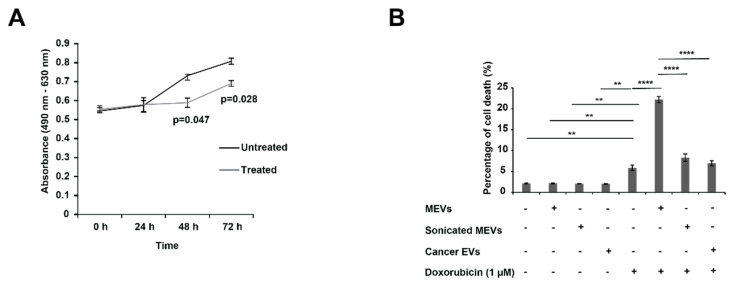
MEVs treatment sensitised aggressive neuroblastoma (NBL) cells to doxorubicin. (**A**) The relative proliferation rate of NBL cells were analysed in the presence and absence of MEVs (100 μg/mL) using MTS assay. (**B**) FACS cell death assay on SK-N-BE2 cells treated with MEVs (100 µg/mL), sonicated MEVs (100 µg/mL), C26 cancer cell-derived EVs (100 µg/mL), doxorubicin (1 µM) or combinatorial therapy. There was a significant increase in percentage of cell death upon the combinational treatment of doxorubicin and MEVs. EVs isolated from cancer cells did not sensitise NBL cells to doxorubicin treatment. Error bars represent the standard error of mean, n = 3, ** denotes the significance of *p* < 0.01, **** denotes the significance of *p* < 0.0001 as determined by *t*-test and one-way analysis of variance (ANOVA) with Turkey–Kramer multiple comparison post-hoc test using GraphPad Prism 8 software.

Next, the anticancer properties of MEVs on NBL cells was investigated using an MTS assay. As a representative of high-risk NBL, SK-N-BE2 cells were used in this study due to their N-Myc amplification status. As shown in Figure 2A, the SK-N-BE2 cells incubated in the presence of MEVs (100 μg/mL) showed a reduction in proliferation at 48 and 72 h (*p* < 0.05). Prior to selection of 100 μg/mL concentration of MEVs, cancer cells were treated with MEVs ranging 20–200 μg/mL, and 100 μg/mL was considered as the optimal concentration (data not shown). Taken together, the above result suggests that bovine MEVs can reduce the metabolic activity of SK-N-BE2 cells.

### 3.3. Treating N-Myc Amplified NBL Cells with MEVs Sensitises the Cells to Doxorubicin

To understand whether MEVs could induce cell death in NBL cells, SK-N-BE2 cells were treated with MEVs (100 µg/mL) and standard of care chemotherapeutic used in treating NBL patients, doxorubicin (1 µM), for 48 h. As shown in Figure 2B, MEVs alone did not induce cell death in NBL cells. Consistent with the literature, doxorubicin did not induce high percentage of cell death (2% for untreated vs. 7% with doxorubicin) in N-Myc amplified SK-N-BE2 NBL cells, though was able to induce significant cell death [41]. However, combinatorial treatment of MEVs and doxorubicin exhibited a five-fold increase in percentage of cell death in NBL cells. To understand whether intact MEVs are required to increase the sensitivity of NBL cells to doxorubicin, MEVs were subjected to sonication. However, the sonicated MEVs in combination with doxorubicin did not increase the sensitivity of NBL cells compared to doxorubicin treatment (Figure 2B). This observation suggests that the MEVs need to be intact and taken up by cells for the EVs to alter the sensitivity of NBL cells. This was further confirmed using MTS assay, where the sonicated MEVs did not decrease the proliferation rate of SK-N-BE2 cells significantly (Figure S1). Next, to confirm that only MEVs could sensitise NBL cells, the cells were treated with EVs isolated from C26 colon cancer cells as a control. There was no change in cell death in the presence of combinatorial treatment of doxorubicin and C26 colon cancer cell-derived EVs compared to doxorubicin alone treatment (Figure 2B). Taken together, these observations suggest that MEVs sensitizes aggressive NBL cells and increases the efficacy of standard-of-care chemotherapeutic drug doxorubicin.

### 3.4. Proteomic Analysis of Neuroblastoma Cells Treated with Bovine Milk-Derived Extracellular Vesicles

**Figure 3:**
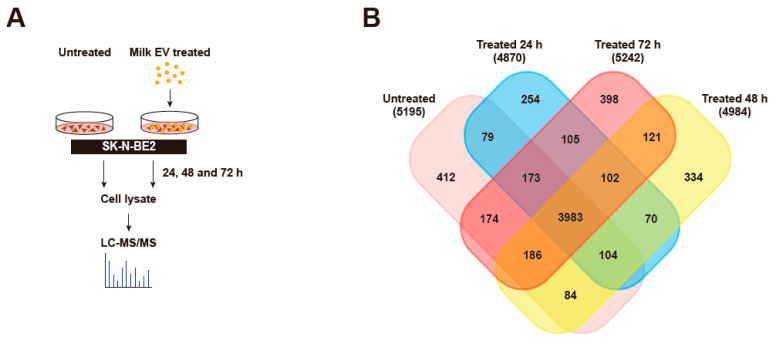
Proteomic analysis of cells treated with MEVs. (**A**) Schematic diagram of sample preparation from label-free quantitative proteomic analysis. (**B**) Venn diagram of proteins distributed between untreated, 24, 48 and 72 h MEVs (100 μg/mL) treated SK-N-BE2 cells. n = 3 biological replicates.

To investigate the proteins and pathways that may contribute to the sensitivity of SK-N-BE2 cells, a temporal proteomic analysis of SK-N-BE2 cells treated with MEVs (100 μg/mL) at varying time points was performed (Figure 3A). Proteins from an equal amount of cell lysate (30 μg) were subjected to label-free quantitative proteomics analysis using LTQ Orbitrap Elite mass spectrometer. With a false discovery rate of <1% and with at least 2 peptides identified in a sample, a total of 5195 proteins were identified in untreated SK-N-BE2 cell lysates. As expected, several proteins were differentially abundant upon MEV treatment (Figure 3B). However, a majority of the proteins (3983) were common between the untreated and MEV treated (24, 48 and 72 h) cell lysates (Figure 3B). There were 412 proteins that was uniquely present in untreated SK-N-BE2 cells compared to the treated cells. Similarly, there were 254, 334 and 398 proteins that were unique to SK-N-BE2 NBL cells treated with MEVs for 24, 48 and 72 h, respectively.

**Figure 4:**
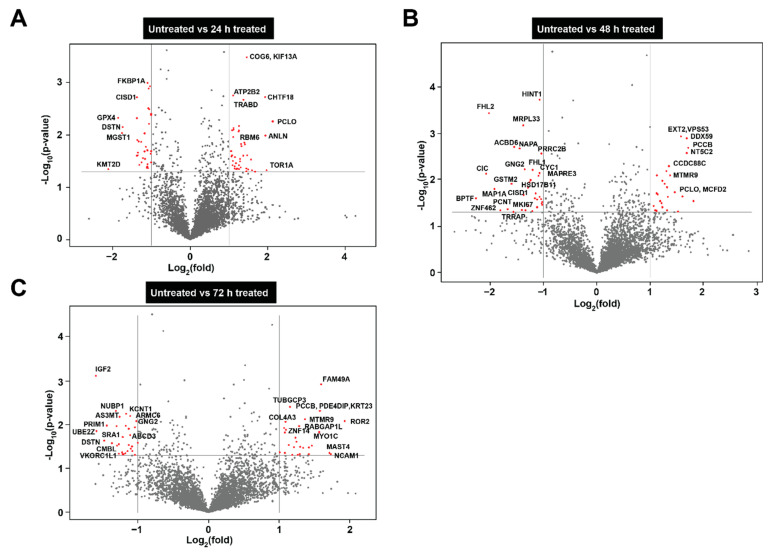
Depiction of differentially abundant proteins via volcano plots. (**A**) Volcano plot depicting the differential abundance of proteins in 24 h treated NBL cells compared to untreated. (**B**) Representation of differential abundance of proteins in 48 h treated NBL cells compared to untreated. (**C**) Volcano plot of differentially abundant proteins at 72 h. *Y* axis= p-value of the protein expression *X* axis = ±2-fold changed proteins.

Next, to identify the proteins that were differentially abundant in SK-N-BE2 cells upon MEVs treatment, Volcano plots were utilized. As shown in Figure 4A, cells treated with MEVs for 24 h were significantly high in abundance with proteins such as COG6, ATP2B2, CHTF18, PCLO, RBM6, ANLN and TOR1A (Supplementary Table S1). Among these, ANLN and TOR1A have been implicated in cytoskeletal dynamics [42,43]. Similarly, proteins including CISD1, GPX4 and DSTN were depleted in NBL cells treated with MEVs after 24 h. At 48 h, MEV treatment significantly decreased the levels of many proteins including HINT1, FHL1, MAP1A, MKI67 and others. MKI67 has been implicated in proliferation [44] and its downregulation correlates with the MTS results. Western blotting analysis confirmed the reduction of MKI67 in NBL cells upon treatment with MEVs (Supplementary Figure S3). At 72 h, MEV treated cells (Figure 4C) were highly enriched in proteins that play a pivotal role maintenance of Golgi complex (MYO1C) and neural cell adhesion (NCAM1) [45,46]. Whereas in accordance with the MTS results (Figure 2A), proteins such as DSTN [47], GPX4 [48], MKI67 [44], BPTF [49] and NUBP [50], that are known to regulate cell cycle, proliferation and pro-survival ability of cells were found highly abundant in untreated NBL cells compared to MEV-treated cells (Figure 4C).

### 3.5. Bovine Milk-Derived Extracellular Vesicles Treatment Depleted Proteins Involved in Cell Cycle and Growth in Neuroblastoma Cells

**Figure 5:**
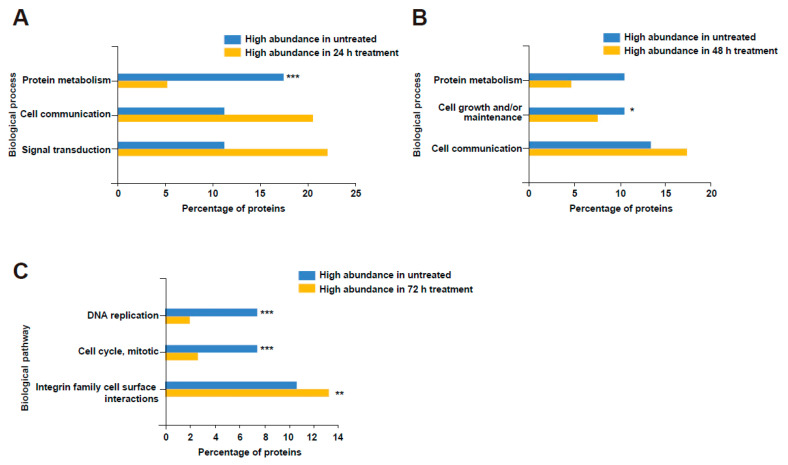
Gene enrichment analysis revealed the enrichment of proteins involved in cellular growth in untreated NBL cells. (**A**) Proteins that are 2-fold differentially abundant in 24 h treated and untreated NBL cells were subjected to enrichment analysis for biological process. (**B**) Proteins that are 2-fold differentially abundant in 48 h treated and untreated NBL cells were subjected to enrichment analysis for biological process. (**C**) Gene enrichment analysis of biological pathways affected by MEV treatment at 72 h. * denotes *p* < 0.05, ** denotes *p* < 0.01 and *** denotes *p* < 0.001 as determined by hypergeometric test.

Next, to further understand the biological processes and pathways that are dysregulated in NBL cells upon MEV treatment, the proteomic data were analysed using FunRich [51]. Proteins that were two-fold abundant in each time point were compared against the proteins that were two-fold abundant in untreated cells. Gene enrichment analysis depicted an increase in the abundance of proteins involved in cell communication and signal transduction in NBL cells upon treatment with MEVs (Figure 5). Proteins implicated in metabolism, DNA replication, cell growth and cell cycle were significantly depleted in NBL cells treated with MEVs at 24 h.

### 3.6. Treatment of Bovine Milk-Derived Extracellular Vesicles Did Not Change the Abundance of Proteins Contributing to Neuroblastoma Aggressiveness

Next, to understand the effect of MEVs on the proteins that play a crucial role in driving NBL aggressiveness, a heatmap was generated (Figure S1). The quantitative expression levels of proteins regulating NBL aggressiveness [52] were examined in the four treatment groups. Even though the treatment of MEVs sensitized NBL cells to doxorubicin, a profound change was not detected in the abundance of proteins known to regulate NBL aggressiveness. Among the proteins, ATRX was depleted upon MEV treatment. The tumour suppressor RRM2 [53] was slightly upregulated in 48 h treatment whereas Rac1, a protein contributing to invasion [54], was less abundant in NBL cells treated with MEVs for 72 h.

### 3.7. Treatment of Bovine Milk-Derived Extracellular Vesicles Induced Cellular Senescence in Neuroblastoma Cells

**Figure 6:**
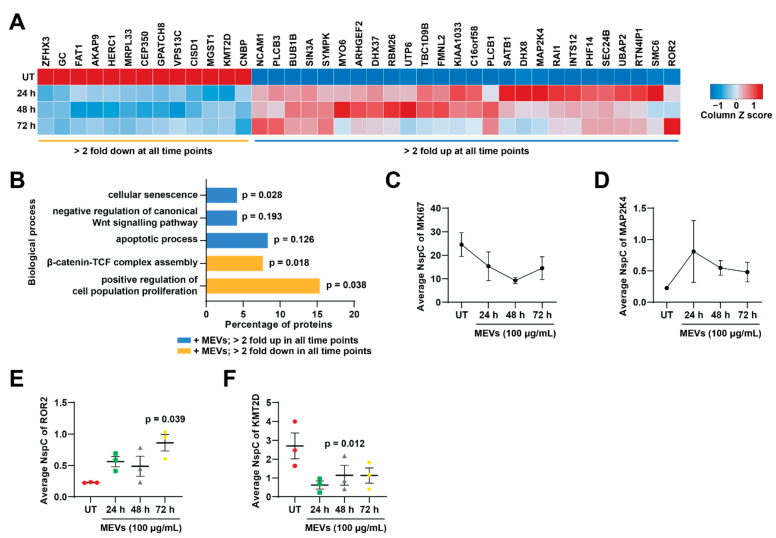
MEV-treated NBL cells were enriched with proteins involved in cellular senescence. (**A**) Heatmap representing the average abundance of proteins that are 2-fold differentially abundant in all the MEV treatment samples compared to the untreated. (**B**) FunRich gene enrichment analysis for biological processes altered due to MEV treatment. (**C**) Average spectral count of MKI67 at different treatment times. (**D**) Normalized spectral count of MAP2K4 upon MEV treatment. (**E**) ROR2 was significantly upregulated in 72 h MEV-treated cells compared to untreated. (**F**) Wnt activator KMT2D was downregulated upon MEV treatment. n = 3-*p*-vales are determined by *t*-test.

Next to identify proteins that are consistently two-fold up or down regulated in all three MEV-treated samples compared to untreated, a heatmap was generated (Figure 6A). The average abundance value of the three-biological replicates for each treatment group was obtained from the proteomics list. Proteins such as FAT1, CNBP, CISD1 and HERC1 were highly depleted upon MEV treatment at 24, 48 and 72 h, whereas proteins such as BUB1B, DHX37, SMC6 and RAI1 were upregulated at all time points after MEV treatment in NBL cells.

In order to understand the biological processes that these proteins are involved in, gene enrichment analysis was performed on proteins that are 2-fold up- or down-regulated in all MEV treatments compared to untreated (Figure 6B). In agreement with the earlier proliferation assay and gene enrichment analysis, cell proliferation was greatly impaired in NBL cells upon MEV treatment. On the contrary, proteins implicated in cellular senescence and apoptotic process were enriched in NBL cells upon MEV treatment. Interestingly, proteins involved in Wnt signalling pathway were also affected upon MEV treatment, where proteins involved in negative regulation of Wnt signalling pathway were upregulated upon MEV treatment. Proteins in β-catenin-TCF complex assembly were in high abundance in untreated NBL cells suggesting Wnt signalling pathway was activated in untreated SK-N-BE2 cells.

Next, to confirm the changes in obtained biological processes, the average NspC values were plotted for some of the proteins that is known to contribute in proliferation, apoptosis and Wnt signalling pathways. Proliferation regulators MKI67 (Figure 6C) and MAP2K4 (Figure 6D) were altered upon MEV treatment [44,55]. Apoptosis regulator ROR2 [56] was significantly upregulated in 72 h MEVs treated cells compared to untreated (Figure 6E). ROR2 is also known to participate in negative regulation of Wnt signalling activity. This observation was further confirmed by the depletion of KMT2D (Figure 6F), a protein involved in Wnt signalling pathway [57], in MEV-treated NBL cells. Overall, these results suggest that the treatment of NBL cells with MEVs attenuates proliferation and Wnt signalling pathway while inducing senescence.

## 4. Discussion

Like many body fluids, milk contain EVs that have shown to impart a role in regulating the signalling pathways of the recipient cells [58]. Over the past few years, there have been immense interest in studying MEVs due to their ability to survive in harsh conditions such as the human gut [16,18]. As a result, the MEVs can be delivered orally and are thought to preserve their cargo including exogenous drugs [59]. NBL is the most common solid tumour in infants [60]. Among the biological factors deciding the aggressiveness of this disease, N-Myc amplification is crucial. Even with the advances in treatment strategies, the survival rate of high-risk NBL is very low. Hence, there is a need for new therapies in sensitizing aggressive NBL. In this study, for the first time, we have demonstrated the temporal proteome changes that occur in NBL cells upon treatment with MEVs and the utility of MEVs as a potential strategy to treat high-risk NBL patients.

The isolated MEVs are heterogenous and were larger in size than the typical 30–150 nm sized EVs isolated from other types of mammalian cells. However, a similar result was observed by li et al., whilst characterizing bovine MEVs [61]. Interestingly, MEVs decreased the proliferative effect of aggressive NBL cells. More importantly, combinatorial treatment of MEVs and doxorubicin significantly increased the percentage of cell death of NBL cells. It must be noted that sonicated MEVs that had lost the membrane integrity were unbale to induce anti-proliferative effect on NBL cells. Similarly, sonicated MEVs were unable to increase the sensitivity of NBL cells to doxorubicin. These data clearly suggest that the membrane of EVs need to be intact and only on uptake of EVs NBL cells exhibit reduced proliferation and enhanced chemosensitivity. In addition to treatment resistance, NBL patients often suffer with significant side effects from prolonged exposure to harsh chemotherapies. Increasing the efficacy of these drugs by combining them with molecules or EVs can improve the survival rates of high-risk NBL patients and enhance the quality of life. Hence, bovine MEVs can be used in combination with chemotherapeutic drugs for therapeutic applications via various routes as shown by Ramesh Gupta and colleagues [23]. The study suggested that oral or IP-based administration of MEVs with anti-cancer drugs reduce tumour burden in xenograft models. It must be noted that the authors reported no change in the plasma cytokine profiles in mice administered orally with bovine MEVs [23]. Consistent with this observation, oral administration of bovine MEVs did not alter the profile of circulating or tumour infiltrating immune cells in pre-clinical syngeneic breast cancer models (Samuel et al. In press). Overall, these data suggest that MEVs may have less immunological properties and can be utilised as potential drug delivery vehicles in therapeutic applications in a context dependent manner.

As another objective of this study was to profile and understand the proteomic changes that occurs in NBL cells treated with MEVs, temporal quantitative proteomics analysis was performed. Treatment with MEVs induced the reduction of proteins implicated in proliferation and increased the abundance of proteins implicated in cellular senescence and apoptosis. Taken together, this study confirms the anti-proliferative properties of MEV suggesting a new therapeutic strategy to target high-risk NBL tumour growth and chemoresistance.

## Data Availability

Proteomics data is contained within the Supplementary Materials.

## Supplementary Materials

The following are available online at https://www.mdpi.com/article/10.3390/cells10040750/s1, Figure S1: Sonicated MEVs did not decrease the proliferation rate of NBL cells; Figure S2: Heatmap of proteins that are crucial in NBL aggressiveness; Figure S3: Validation of proteomics analysis using Western blotting; Table S1: List of identified proteins.
