# Supplementary material for: Temporal Quantitative Proteomics Analysis of Neuroblastoma Cells Treated with Bovine Milk-Derived Extracellular Vesicles Highlights the Anti-Proliferative Properties of Milk-Derived Extracellular Vesicles
Source: Cells. 2021 Mar 29;10(4):750. doi: 10.3390/cells10040750 (PMC8065825; doi:10.3390/cells10040750)
Supplement: Supplementary file 1 [file cells-10-00750-s001.zip › cells-1140147-supplementary/cells-1140417 - Suppl Figure.pdf]

Temporal Quantitative Proteomics Analysis of Neuroblastoma Cells Treated with Bovine Milk-Derived Extracellular Vesicles Highlights the Anti-Proliferative Properties of Milk-Derived Extracellular Vesicles

Pamali Fonseka [1,*] Taeyoung Kang [1], Sing Chee [1], Sai V. Chitti [1], Rahul Sanwlani [1], Ching-Seng Ang [2] and Suresh Mathivanan [1,*]

Supplementary Figure:

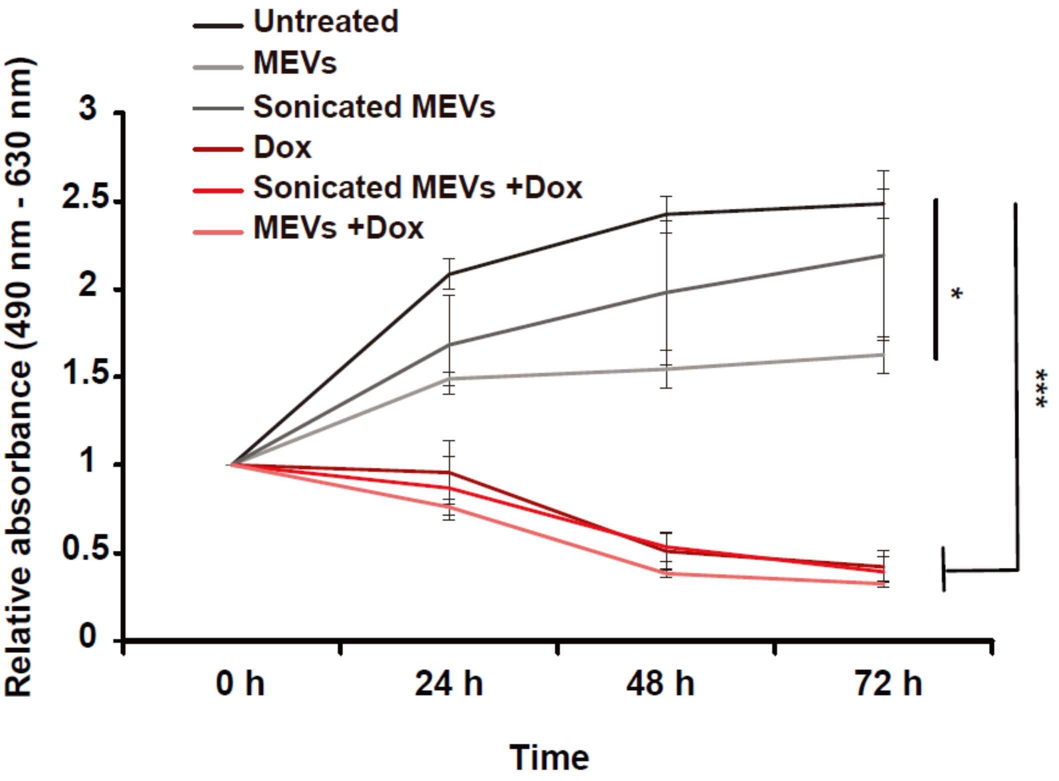

Figure S1. Sonicated MEVs did not decrease the proliferation rate of NBL cells. The relative proliferation rate of NBL cells were analyzed in the presence and absence of MEVs (100 μg/mL) or sonicated MEVs (100 μg/mL) or combinatorial treatment using MTS assay. Doxorubicin and combinatorial treatment of doxorubicin and MEVs or sonicated MEVs decreased proliferation rate of SK-N-BE2 cells significantly. Error bars represent the standard error of mean, n = 3, * denotes the significance of $p < 0.05$, ** denotes the significance of $p < 0.01$, **** denotes the significance of $p < 0.0001$ as determined by t-test and one-way analysis of variance (ANOVA) with Turkey-Kramer multiple comparison post-hoc test using GraphPad Prism 8 software

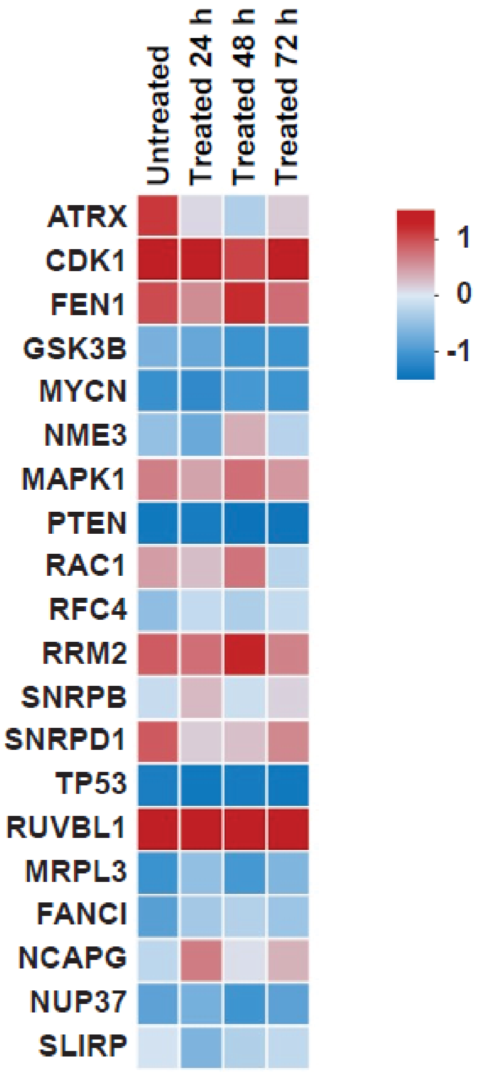

Figure S2. Heatmap of proteins that are crucial in NBL aggressiveness. Heatmap was generated using the quantitative abundance of proteins involved in NBL aggressiveness.

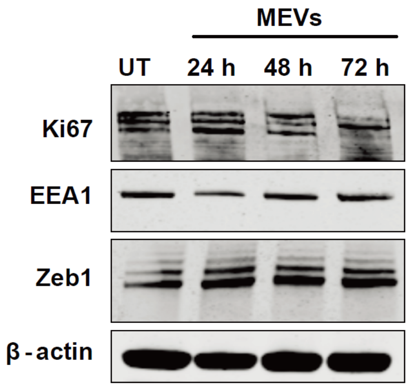

Figure S3. Validation of proteomics analysis using Western blotting. Ki67 and EEA1 expression levels were decreased upon treatment of MEVs. Zeb1 protein expression levels were increased upon treatment with MEVs in SK-N-BE2 cells. Here, β-actin was used as the loading control.
